# Jujube mucilage as a potential stabilizer in stirred yogurt: Improvements in the physiochemical, rheological, and sensorial properties

**DOI:** 10.1002/fsn3.1230

**Published:** 2019-10-10

**Authors:** Mandana Yekta, Sara Ansari

**Affiliations:** ^1^ Department of Food Science and Technology Islamic Azad University Kazerun Iran

**Keywords:** jujube mucilage, physiochemical properties, rheological properties, sensory attributes, stirred yogurt

## Abstract

Here, the mucilage of jujube was extracted and used as a natural stabilizer in the production of stirred yogurt. Yogurts were enriched with different concentrations of jujube mucilage (i.e., 0, 0.1, 0.15, and 0.2%), and their physical, chemical and sensory attributes were analyzed during 21 days of storage at 4°C. The results showed that the protein and fat contents of the yogurts were not significantly different compared with each other, while higher ash contents were obtained in yogurts which contained higher concentrations of the mucilage. The acidity and proteolysis of the stirred yogurts were enhanced in the presence of mucilage, and they exhibited lower concentrations of diacetyl and acetaldehyde, although the differences were not significant among the samples of different treatments. The storage time had adverse and direct effects on the amounts of acetaldehyde and diacetyl, respectively. The effects of storage time and the presence of jujube mucilage in yogurts caused a significant decrease in the percentage of syneresis, while their viscosity and WHC values increased. The magnitudes of dynamic moduli (G, *G''*), complex viscosity (η*), and loss tangent (tan δ) of stirred yogurts increased by increasing the concentration of jujube mucilage. The yogurts which had been enriched with mucilage were preferred slightly less by tasters during the storage period, but these differences did not amount to a statistical significance. Generally, the results of the present study showed that the jujube mucilage can be potentially used as a natural stabilizer in stirred yogurt.

## 1 INTRODUCTION

Yogurt is a well‐known, fermented, milk‐based product which is consumed in many parts of the world. It usually exerts functional effects on the body and on the many metabolisms involved in the gastrointestinal tract (GIT), especially in the large intestine. Optimum consistency and stability are two major qualities in yogurts. Commercial producers seek higher levels of total soluble solids in yogurts, along with the control of the starter culture and other processing variables, which can improve the consistency of yogurts (Khalifa, Elgasim, Zaghloul, & Mahfouz, 2011). Recent attention has been directed at natural modifiers such as starch, gum Arabic, Persian gum, carboxymethyl cellulose (CMC), guar gum, and xanthan in dairy products, and their applications have gained scientific developments (Dabestani, Kadkhodaee, Phillips, & Abbasi, 2018; Liu, Wang, Liu, Wu, & Zhang, 2018). These hydrocolloids can serve as gelling or thickening agents, thereby stabilizing the yogurt matrix and increasing the viscosity. The hydrocolloids inhibit syneresis, preserve the yogurt structure, and change the mouthfeel. Meanwhile, they are incorporated into dairy products in order to affect their rheological, structural, and sensorial characteristics.

One group of natural additives is the mucilage. As a heterogeneous polysaccharide complex, it can turn into slimy masses when water is added to it. Different forms of mucilage are obtained mainly from plant parts such as seeds in addition to specific microorganisms and marine algae. Generally, plant‐derived types of mucilage are historically recognized for their medicinal applications. Food industries can take an advantage of them as water‐retention agents, thickeners, emulsion stabilizers, suspending agents, gelling agents, film former binders, and sustained‐release agents (Hassan et al., 2015).

Jujube *(Ziziphus* spp.) is a fruit which is widely cultivated in tropical and subtropical regions such as in southern and eastern Asia, Australia, and Europe. In traditional Chinese medicine, the *Z. jujuba* fruit has been considered as one of the five most valuable fruits. Over the past several decades, *Z.* *jujube* has been recognized as a source of sedative compounds with hepatoprotective effects, and they are also characterized by antioxidant activities, compounds with immunological properties and anti‐inflammatory effects (Ji et al., 2017; Wojdyło, Carbonell‐Barrachina, Legua, & Hernández, 2016). The diverse array of its pharmacological effects has roots in the richness of its chemical ingredients, that is, phenolic acids (benzoic acids and hydroxycinnamic acids), vitamin C, flavonoids (flavonols and flavan‐3‐ols), nucleosides, triterpenic acids, and pigments (particularly anthocyanin compounds), along with an abundance of mucilage or polysaccharides that concern the interests of the current study (Ji et al., 2017; Wojdyło et al., 2016; Xie, Tang, Jin, Li, & Xie, 2016). The *Z. jujube* fruit is particularly rich in mucilage as it constitutes a large group of the biologically active compounds in the fruit (Ji et al., 2017). Mucilage can be considered as specific forms of complex polysaccharides which are mostly branched by l‐rhamnose, l‐arabinose, d‐glucose, d‐xylose, and galacturonic acid (Thanatcha & Pranee, 2011). Different varieties of the *Z. jujuba* fruits exhibit various molecular weights for their juice which range from 10^4^ to 10^6^ Da and can be recorded under diverse experimental conditions (Ji et al., 2017). Thanatcha and Pranee (2011) reported a comparative study on the characteristics of mucilage obtained from the jujube fruit and similar features of the guar and xanthan gum. It was reported that the jujube mucilage exhibited lower levels of water‐holding capacity and emulsion capacity, but higher amounts of oil absorption, as compared to the guar and xanthan gum. This pseudoplastic hydrocolloid, that is, the jujube mucilage, can be employed to increase the stability and viscosity of food materials.

Due to the numerous benefits of jujube mucilage and its similar function with some types of gum, there has been more interest to study this mucilage as gum substitute in food products. Here, the aim is to evaluate the possibility of using the jujube mucilage as a stabilizer in stirred yogurt and to examine its functions in relation to the physicochemical, rheological, and sensory properties of yogurt.

## 2 MATERIALS AND METHODS

### 2.1 Extraction of mucilage from jujube powder

Jujube fruits were purchased from a local market. Impurities on the fruits were cleaned off. Then, the fruity pulp was separated, squashed, and mixed using a blender (IKA). The fruits were stored at 4°C before the extraction of mucilage which followed a method proposed by Thanatcha and Pranee (2011). To describe the procedure briefly, 200 ml of distilled water was added to 2.0 g of the jujube powder. The mixture was heated and rotated for 20 min (at 100°C and 400 rpm) with a magnetic stirrer (Heidolph). After the separation of waste materials by filtration, which was performed by a fine cloth, accompanied by a Büchner funnel and the process of centrifugation (Universal, Germany) at 3800g for 15 min, the mucilage solution or supernatant liquid was obtained and precipitated in ethanol (with a ratio of 1:4). The solution was then stored at 4°C for 24 hr. The obtained mucilage was filtered through a clean cotton cloth and dried into powder after 48 hr of storage at ambient temperature. The dried mucilage was stored at 4°C for further analysis and for use in the stirred yogurt.

### 2.2 Yogurt production

The milk, which was used for the production of yogurt, contained natural amounts of fat and soluble solid content. It was mixed with the jujube mucilage powder at different concentrations, that is, 0% as the control (JM 0), 0.1% (JM 0.1), 0.15% (JM 0.15), and 0.2% (JM 0.2). Then, the milk was homogenized at 60°C and later pasteurized in a water bath at 90–95°C for 3–5 min. After cooling down to 40–45°C, the milk was inoculated with 2.5% (w/v) of the lyophilized mixed starter cultures (Lactobacillus bulgaricus and Streptococcus thermophiles, Mediterranea Biotecnologie srl., Termoli, Italy) (1:1) and incubated at 40–45°C to complete the coagulation. When the pH reached 4.6, the gel samples were stirred gently for 2 min. All of the stirred yogurts were stored at 4°C until the experiments were performed. The yogurts were analyzed at regular intervals, that is, after 1, 7, 14, and 21 days following the start of the experiment.

### 2.3 Chemical analysis

After the first day of preparation, the stirred yogurts were analyzed for protein, fat, and ash contents (AOAC, 1995). The changes in pH of the stirred yogurts during storage were measured using a glass electrode laboratory pH meter (SOMET CZ, USA). The titratable acidity was determined (as lactic acid %) according to the titration procedure (AOAC, 1995). The extent of proteolysis in the yogurt samples during the storage was calculated according to the ratio of soluble nitrogen/total nitrogen (SN/TN) (Hassan et al., 2015). The fluctuations in the concentrations of acetaldehyde and diacetyl in the yogurt samples were measured using a spectrophotometer (NETZSCH) as described in the available literature (Hassan et al., 2015).

### 2.4 Physical analysis

#### 2.4.1 Whey separation

The volume of whey on the surface of yogurt samples is generally considered as the wheying‐off segment (ml/100 g yogurt), and here, it is measured according to the siphon method described by Amatayakul, Halmos, Sherkat, and Shah (2006). In order to quantify the whey separation, 25 g of yogurt samples was poured into a funnel lined with a Whatman filter paper number 41. The whey (ml) was collected after 1 hr of drainage at 4°C, and its quantity was expressed as an index of whey syneresis.

### 2.5 Water‐holding capacity (WHC)

To measure the water‐holding capacity, 5 g of yogurt samples was poured into centrifuge tubes and the centrifugation was conducted at 3885 g for 30 min at 10°C. Then, the supernatant was separated and the sediment was weighed. Eventually, the WHC was calculated by the following equation (Sahan, Yasar, & Hayaloglu, 2008):

(1) $$\text{\textbackslash{}\% WHC}=1-\frac{\text{weight of sediment obtained from centrifuge}}{\text{Initial weight of yogurt samples}}$$

### 2.6 Apparent viscosity

During storage, the apparent viscosities of the samples were measured by a rotational Brookfield digital viscometer (Model DV‐II) equipped with a spindle‐LV4. The experiments were conducted under stable conditions: a shear rate of 30 rpm at 30 s and at a temperature of 10°C.

### 2.7 Dynamic rheological properties

The dynamic viscoelastic properties of the gel‐like structure of yogurt samples were characterized on the first day of storage using a Physical MCP300 rheometer (Anton Paar), accompanied by a cone‐plate geometry (50 mm diameter and 1.00 mm gap) at 15°C. Strain sweeps (i.e., the amplitude tests) were performed in order to determine the linear viscoelastic region at a constant frequency of 1.0 Hz while the strain (γ) varied from 0.1% to 100%. The frequency sweeps were carried out at a constant strain of 1%, as the frequency increased from 0.1 to 12 rad/s (0.03 to 20 Hz) and led to the specification of the storage modulus (*G*'), the loss modulus (*G''*), the complex module (G*), and the loss tangent which is defined as tan δ = (*G''*/*G*'). Furthermore, the complex viscosity (η*) is an indicator of the stiffness of a material which could be achieved by the ratio of the complex module to the frequency (ω) (Ramirez‐Santiago et al., 2010). All of the dynamic rheological measurements were conducted in triplicate, and the reported results were expressed as an average of the three measurements.

The following models specified the descriptions of the dynamic rheological curves of the yogurt samples:

(2) $$G^{{}^{\prime }}=a\omega^{b}$$

(3) $$G^{{}^{\prime \prime }}=c\omega^{d}$$

where the rheological behavior is characterized by *a*, *b*, *c*, and *d* as parameters.

(4) $$\eta^{*}=k^{*}\omega^{n*-1}$$

where n* (or *b*) is the dynamic power law factor, k* (or *a*) is the dynamic consistency index (Pa sn*), ω is the radial frequency (s − 1), and η* is the dynamic viscosity (Pa sn*−1). The system is elastic when n* = 0, and the decrease in η* is parallel to the increase in the value of ω. A viscous system is described by n* = 1 and by the stability of the η* value. When the system is viscoelastic, the value of n* ranges from 0 to 1.

### 2.8 Color

The evaluation of color or hues among the yogurt samples during storage involved the use of a colorimeter (Minolta, CR‐300) which was initially calibrated by black and white surfaces. Based on the light reflection, the parameters of measurement were defined as L* (lightness), a* (redness and greenness), and b* (yellowness and blueness) which described each aspect of color.

### 2.9 Sensory analysis

Sensory evaluation was conducted according to recommendations described in ISO 13,299:2003. The sensory panel consisted of 10 persons (five male and five female, aged from 25 to 45) who were trained how to evaluate the samples according to the 5‐point hedonic test. The organoleptic properties of yogurt samples during storage were examined in terms of appearance, consistency, odor, flavor, and overall acceptance. The maximum score was considered as 5, which indicated the best possible quality of a yogurt sample, and the least score was defined as 1, representing the worst quality in a sample (Hosseini & Ansari, 2019).

### 2.10 Statistical analysis

All experiments were performed in triplicate, and data analysis was conducted by the SPSS V.18 software. To study the variables and their effects, a complete randomized design was used. The analysis of variance (ANOVA) was performed, and the comparison of mean values was accomplished by Duncan's multiple range test (*p* ≤ .05).

## 3 RESULTS AND DISCUSSION

### 3.1 Chemical composition

The average chemical composition and the relevant variations in the stirred yogurt are presented in Table 1. Adding jujube mucilage was observed to have no significant effect on the fat and protein content of yogurt samples. However, the addition of high levels of jujube mucilage to yogurt increased the ash content, which can be due to the presence of minerals in the jujube mucilage. The results were in agreement with those reported by Hassan et al. (2015) and Sahan et al. (2008) where adding guar gum at a concentration of 0.1% and β‐glucan at concentrations of 0.25 to 1% did not result in significant differences in the fat and protein contents. However, the ash content of free‐fat yogurt samples increased along with the increase in the beta‐glucan content. Lobato‐Calleros, Ramírez‐Santiago, Vernon‐Carter, and Alvarez‐Ramirez (2014) reported similar results for yogurt samples prepared with native and chemically modified maize starch, and tapioca starch.

**Table 1:** Chemical composition of yogurt samples containing different concentrations of jujube mucilage (mean ± *SD*)*

Treatments	Protein (%)	Fat (%)	Ash (%)
JM 0	2.91 ± 0.01^a^	2.58 ± 0.01^a^	0.70 ± 0.00^b^
JM 0.1	2.83 ± 0.01^b^	2.57 ± 0.02^a^	0.75 ± 0.05^b^
JM 0.15	2.85 ± 0.01^b^	2.57 ± 0.01^a^	0.75 ± 0.05^b^
JM 0.2	2.83 ± 0.01^b^	2.57 ± 0.01^a^	0.90 ± 0.00^a^

* Different superscript letters indicate significant differences within the columns (*p* < .05).

### 3.2 pH and titratable acidity

There were changes in the titratable acidity (%) and pH of yogurt during the 21 days of storage at 4°C (Table 2). The addition of jujube mucilage to yogurts led to a significant increase in the titratable acidity. The yogurt samples with different concentrations of mucilage did not show significant differences in this regard (*p*> .05). In addition, as presented in Table 2, over time, either in the control sample or in the samples enriched with mucilage, titratable acidity was significantly increased. On the other hand, Table 2 shows that the trend of change in the pH was opposite to that of titratable acidity. The pH of the samples decreased significantly by adding jujube mucilage. As shown in Table 2, on the first day of storage, there was no significant difference between the samples which contained 0.1 and 0.15% mucilage (*p*> .05). These changes were highlighted as the storage reached the seventh day, and thereafter, the changes in the pH values showed significant differences, regarding the 0 and 0.1% mucilage treatments. On the other hand, the control sample exhibited significant changes in the pH during storage just before the seventh day, and the pH values of the remaining days did not show significant differences (*p*> .05). However, for the yogurt sample with 0.1% mucilage, significant changes were observed in the pH throughout the 21 days of storage. Meanwhile, other concentrations of mucilage caused changes that were similar to the control sample, that is, with significant values before the seventh day (Table 2). Lactic acidification mainly determines the value of acidity in yogurts produced by conventional formulations. Lactic acidification proceeds the culture of the starter bacteria which progressively convert lactose to lactic acid during storage. However, the acidity of yogurts may increase slightly more than usual when other mechanisms are activated by the addition of the jujube mucilage. Indeed, it can be stated that jujube mucilage due to the polysaccharide structure as well as its prebiotic properties can increase the activity of yogurt starter and consequently increase acid production and decrease pH. Relevantly, a previous study claimed that probiotic bacteria can survive and grow for longer durations when fermented milks are enriched with citric fiber, thereby contributing to a faster conversion of lactose into lactic acid (Sendra et al., 2010). Another report suggested that the presence of a highly branched, acidic arabinoxylan in the psyllium husk gum (PHG) structure was a possible cause of the higher acidity observed in yogurts enriched with PHG (Ladjevardi, Gharibzahedi, & Mousavi, 2015). This is also confirmed by a previous study on the conditions of stirred yogurt when the samples were enriched with a type of soluble dietary fiber obtained from *Pachyrhizus erosus*. After 14 days of storage, there was a significant increase in the acidity of yogurt samples that had been enriched with the dietary fiber (Ramirez‐Santiago et al., 2010). Similar observations were found in soy‐yogurt samples incorporated with different stabilizers including maize starch, cassava starch, and gelatin. It was reported that the inclusion of stabilizers in the soy yogurt led to the decrease in pH, as a result of the starter microorganisms which metabolize sugars into acids (Jimoh & Kolapo, 2007). Contrary to our results, Hassan et al. (2015), Sahan et al. (2008), and Nikoofar, Hojjatoleslami, and Shariaty (2013) reported no remarkable changes in the pH and acidity values of yogurt after it was enriched with guar gum (0.1%)/ β‐glucan (0.25%–1%) and quince seed mucilage at concentrations of 0.03, 0.05, and 0.1%, respectively.

**Table 2:** Chemical characteristics of yogurt samples during 21 days of storage at 4*°C* (mean ± *SD*)*

Treatments	Storage time (days)			
	1	7	14	21
Titratable acidity (%)				
JM 0	0.96 ± 0.00^Bd^	1.45 ± 0.014^Cc^	1.63 ± 0.03^Bb^	1.97 ± 0.01^Aa^
JM 0.1	1.06 ± 0.00^Ad^	1.54 ± 0.01^ABc^	1.71 ± 0.01^Ab^	1.97 ± 0.06^Aa^
JM 0.15	1.06 ± 0.00^Ad^	1.56 ± 0.01^Ac^	1.72 ± 0.01^Ab^	1.98 ± 0.01^Aa^
JM 0.2	1.06 ± 0.00^Ad^	1.52 ± 0.00^Bc^	1.73 ± 0.01^Ab^	1.97 ± 0.02^Aa^
pH				
JM 0	4.07 ± 0.01^Aa^	3.72 ± 0.01^Ab^	3.71 ± 0.01^Ab^	3.70 ± 0.02^Ab^
JM 0.1	3.96 ± 0.01^Bb^	3.70 ± 0.01^ABb^	3.67 ± 0.01^Bc^	3.66 ± 0.01^Bd^
JM 0.15	3.95 ± 0.01^Ba^	3.68 ± 0.01^BCb^	3.66 ± 0.02^Bb^	3.66 ± 0.01^Bb^
JM 0.2	3.90 ± 0.01^Ca^	3.67 ± 0.01^Cb^	3.66 ± 0.01^Bb^	3.66 ± 0.01^Bb^
Proteolysis (%)				
JM 0	8.45 ± 0.07^Ad^	10.35 ± 0.21^Ac^	11.25 ± 0.07^Db^	12.55 ± 0.21^Da^
JM 0.1	8.50 ± 0.14^Ad^	10.70 ± 0.14^Ac^	11.95 ± 0.03^Cb^	13.80 ± 0.04^Ca^
JM 0.15	8.60 ± 0.14^Ad^	10.65 ± 0.07^Ac^	12.30 ± 0.04^Bb^	13.95 ± 0.02^Ba^
JM 0.2	8.50 ± 0.14^Ad^	10.65 ± 0.21^Ac^	12.45 ± 0.07^Ab^	14.35 ± 0.04^Aa^
Acetaldehyde (ppm)				
JM 0	2.26 ± 0.12^Aa^	1.80 ± 0.08^Ab^	1.31 ± 0.04^Ac^	1.10 ± 0.01^Ad^
JM 0.1	2.16 ± 0.04^Aa^	1.19 ± 0.02^Bb^	1.07 ± 0.04^Bc^	0.97 ± 0.05^BCd^
JM 0.15	2.14 ± 0.04^Aa^	1.17 ± 0.10^Bb^	1.05 ± 0.07^Bb^	0.89 ± 0.09^BCc^
JM 0.2	2.10 ± 0.04^Aa^	1.16 ± 0.05^Bb^	1.05 ± 0.08^Bc^	0.85 ± 0.10^Cd^
Diacetyl (ppm)				
JM 0	3.40 ± 0.02^Ad^	7.81 ± 0.03^Ac^	8.89 ± 0.04^Ab^	8.97 ± 0.04^Aa^
JM 0.1	3.31 ± 0.09^ABd^	4.03 ± 0.10^Bc^	4.95 ± 0.09^Bb^	5.12 ± 0.15^Ba^
JM 0.15	3.20 ± 0.08^Bd^	3.98 ± 0.09^Bc^	4.87 ± 0.09^Bb^	5.09 ± 0.07^Ba^
JM 0.2	3.19 ± 0.04^Bd^	3.82 ± 0.02^Bc^	4.84 ± 0.03^Bb^	4.93 ± 0.06^Ba^

* Different lower case letters in rows and different uppercase letters in columns mean significant differences at probability level of 5%.

### 3.3 Flavor compounds

Acetaldehyde, diacetyl, acetone, and acetoin are the most common volatile compounds in yogurt. The typical aroma of yogurt is mostly caused by acetaldehyde, and *Lactobacillus delbrueckii ssp. bulgaricus* is the starter microorganism which creates this aromatic compound (Khalifa et al., 2011). Acetaldehyde is expected to have a concentration ranging from 23 to 41 mg/kg in yogurt, which generates the optimum flavor in yogurt. Furthermore, the researchers claimed that yogurt has a distinctive buttery or butterscotch taste of fermented milk that is caused by diacetyl.

The current research indicated the occurrence of changes in the acetaldehyde and diacetyl contents of yogurt samples during storage (Table 2). As can be seen, in all yogurt treatments, the concentration of acetaldehyde has been reduced significantly (*p* < .05) during the storage time, as the highest and lowest concentrations were observed on the first and 21st days of storage, respectively. Acetaldehyde can evaporate or be converted to ethanol by the action of alcohol dehydrogenase which is produced by yogurt starters, and this can partly explain the decrease in the concentration of acetaldehyde during storage (Sahan et al., 2008). The decrease in the concentration of acetaldehyde during storage was found by Sahan et al. (2008) and Hassan et al., (2015) in yogurt enriched with plant polysaccharides, guar gum, and cress seed mucilage, respectively. As presented in Table 2, the incorporation of mucilage at different concentrations reduced the concentration of acetaldehyde. On the first day, there was no significant difference between the amounts of acetaldehyde in different treatments. However, after 7, 14, and 21 days, the control sample showed the highest concentration of acetaldehyde, while the samples with mucilage showed lower concentrations. These differences were statistically significant (*p* < .05). Moreover, there was no significant difference in the acetaldehyde concentration of samples incorporated with mucilage on any day of storage. In a previous study, low‐fat yogurts which contained 0.2% Slendid® and free‐fat yogurts enriched with waxy maize exhibited similar patterns of changes in the acetaldehyde concentration (Abd El‐Aziz, Ahmed, Sayed, Mahran, & Hamad, 2004; Farahat, 1999). According to Gaafar (1992), who categorizes yogurts in terms of flavor, all the yogurts produced in the present study fall into the category of weak flavored yogurts due to containing < 4.00 ppm acetaldehyde.

As presented in Table 2, the amount of diacetyl in all samples, with or without mucilage, increased during the storage. Accordingly, in each treatment, the highest diacetyl content was observed in any particular sample on day 21, and the lowest content of diacetyl was recorded in the sample on the first day. This difference was statistically significant (*p *< .05). Moreover, higher concentrations of mucilage in yogurts caused a reduction in the diacetyl amount, but there were no significant differences among the samples which contained the mucilage at any specific point of the storage time (*p*> .05). Hassan et al. (2015) demonstrated that the addition of guar gum to yogurts can result in a slight increase in the diacetyl concentration of yogurt samples during the storage time. A similar trend was recorded in low‐fat yogurts which contained waxy maize (Abd El‐Aziz et al., 2004), cress seed mucilage (Gaafar, 1992), guar gum (Hassan et al., 2015), or inulin (Khalifa et al., 2011).

Regarding the results of the current study, it can be stated that the addition of jujube mucilage as a hydrocolloid can reduce the water activity and thus reduce the growth of the starter bacteria. This can reduce the production of flavoring substances such as acetaldehyde and diacetyl.

### 3.4 Proteolysis

The occurrence of changes in the proteolysis of yogurt showed various trends during storage (Table 2). It was observed that during the storage time, the proteolysis rate significantly increased (*p* < .05) within all samples (with or without jujube mucilage). The highest rate was observed on day 21, and the least was ascribed to day 1. The increase in the proteolysis of the samples over time reflects the proteolytic activity of the lactic acid bacteria during the storage period. Plain set‐type yogurt showed a similar trend of increase in the occurrence and extent of proteolysis during storage (Guzel‐Seydim, Sezgin, & Seydim, 2005). Furthermore, low‐fat yogurts which contain exopolysaccharide (EPS)‐producing cultures and those which contain cress seed mucilage or guar gum are reportedly samples that are capable of higher levels of proteolysis (Hassan et al., 2015).

**Figure 1:**
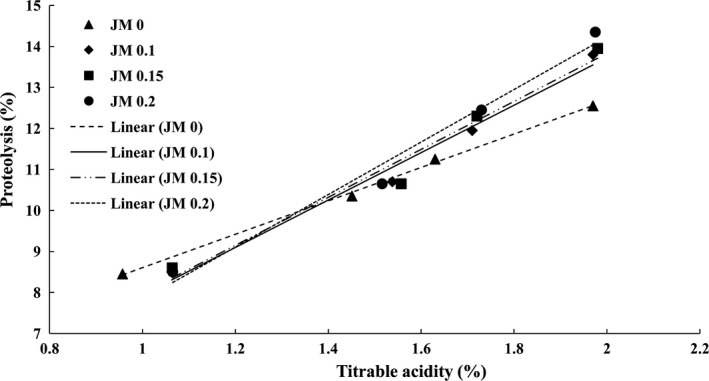
The relationship between the proteolysis and acidity values of yogurt samples during 21 days of storage at 4°C**.** [JM 0: y = 5.84x + 1.14 (*R* ^2^ = 0.97), JM 0.1: y = 5.78x + 2.161 (*R* ^2^ = 0.98), JM0.15: y = 4.07x + 4.53 (*R* ^2^ = 0.99), JM 0.2: y = 6.41x + 1.42 (*R* ^2^ = 0.98)]

Moreover, it was revealed that the jujube mucilage was a cause for an increase in the amount of proteolysis. There was no significant difference between different treatments until the seventh day. However, on days 14 and 21, the degrees of proteolysis in the plain sample (0% mucilage) and in the yogurt which contained 0.2% mucilage were minimum and maximum, which were statistically significant results (*p *< .05) (Table 2). A similar observation was found by Hassan et al. (2015) and Abd El‐Aziz et al. (2004) in yogurt containing cress seed mucilage or guar gum and free‐fat yogurt containing waxy maize. Indeed, the polysaccharide structure of mucilage is reportedly capable of increasing the activity of the starter which, in turn, leads to an increase in the proteolytic activity and a decrease in pH (Donkor, Nilmini, Stolic, Vasiljevic, & Shah, 2007). As shown in Figure 1, a strong positive correlation was found between proteolysis and yogurt acidity in all samples (*R* ^2^> 0.97). Accordingly, the increase in acidity indicates an increase in the activity of the starter and thus an increase in the rate of proteolysis.

### 3.5 Syneresis and water‐holding capacity (WHC)

Whey separation or syneresis is an unfavorable phenomenon which occurs in yogurt. The distinguishable separation of whey from the gel‐like surface of set‐type yogurts can regularly be defined as whey separation. The duration of storage is commonly associated with certain levels of syneresis (Lobato‐Calleros et al., 2014). Supplementing the yogurt with some kind of hydrocolloids or mucilage, due to their ability to absorb and maintain water, can reduce this problem to a great extent (Basiri, Haidary, Shekarforoush, & Niakousari, 2018). As shown in Table 3, syneresis percentages of samples were significantly decreased during the storage period (*p *< .05). Actually, it should be noted that at a lower temperature, stronger bonds are able to be formed between the particles of the gel, and it is possible that their numbers increase. This can be assumed when swollen particles become linked to each other on a broader space. In addition, the inclusion of mucilage significantly reduced the syneresis in this study (*p* < .05) (Table 3). On days 7, 14, and 21, there were significant differences between the control sample (which showed the highest rate of syneresis) and the samples incorporated with jujube mucilage (*p *< .05). However, there was no significant difference between samples containing different concentration of mucilage (*p*> .05). Indeed, jujube mucilage (like flaxseed mucilage) acts as an uncharged hydrocolloid which reduces the syneresis by increasing the viscosity of the continuous phase. Similar reductions in the whey syneresis among yogurt samples have been reported by enriching yogurts with native and chemically modified starches, quince seed mucilage, cress seed mucilage or guar gum, CMC, flaxseed mucilage, and a combination of the mentioned materials (Lobato‐Calleros et al., 2014; Nikoofar et al., 2013); Hassan et al., 2015); Basiri et al., 2018). According to Sahan et al. (2008), the addition of β‐glucan to nonfat yogurts reduced the degree of syneresis. However, there was no relationship between the β‐glucan concentration and the syneresis value. In addition, the value of syneresis on the first day was more than the value on the 15th day. A report suggests that high concentrations of PHG (psyllium husk gum) can be associated with the formation of a strong network structure which can ultimately increase the viscosity, in addition to a slower rate of syneresis in low‐fat yogurt (Ladjevardi et al., 2015).

**Table 3:** Physical characteristics of yogurt samples during 21 days of storage at 4*°C* (mean ± *SD*)*

Treatments	Storage time (days)			
	1	7	14	21
Syneresis (%)				
JM 0	65.37 ± 0.15^Aa^	42.39 ± 0.04^Ab^	38.19 ± 0.13^Ac^	22.56 ± 0.47^Ad^
JM 0.1	64.90 ± 0.02^Aa^	41.15 ± 0.12^Bb^	36.69 ± 0.28^Bc^	21.48 ± 0.27^Bd^
JM 0.15	64.43 ± 0.04^Aa^	40.97 ± 0.08^Bb^	35.84 ± 0.08^Bc^	21.11 ± 0.31^Bd^
JM 0.2	63.13 ± 0.57^Ba^	40.54 ± 0.07^Bb^	36.66 ± 0.37^Bc^	20.47 ± 0.19^Bd^
WHC (%)				
JM 0	31.82 ± 0.39^Cc^	30.83 ± 0.95^Bc^	36.09 ± 1.13^Bb^	41.16 ± 0.54^Ca^
JM 0.1	35.97 ± 0.15^BCd^	38.69 ± 0.41^Ac^	41.99 ± 0.98^Ab^	46.51 ± 0.72^Ba^
JM 0.15	37.07 ± 3.32^ABc^	39.85 ± 2.31^Ab^	41.38 ± 0.13^Ab^	47.30 ± 0.57^ABa^
JM 0.2	41.35 ± 0.67^Ac^	41.88 ± 1.49^Abc^	42.77 ± 0.48^Ab^	48.28 ± 0.35^Aa^
Viscosity (Pa.s)				
JM 0	6.22 ± 0.04^Dc^	5.55 ± 0.05^Cd^	6.48 ± 0.03^Cb^	7.74 ± 0.05^Da^
JM 0.1	6.88 ± 0.09^Cb^	5.58 ± 0.10^Cd^	6.60 ± 0.05^Cc^	9.25 ± 0.04^Ca^
JM 0.15	7.15 ± 0.04^Bc^	6.12 ± 0.02^Bd^	8.41 ± 0.10^Bb^	10.32 ± 0.09^Ba^
JM 0.2	7.48 ± 0.02^Ac^	6.83 ± 0.02^Ad^	8.70 ± 0.05^Ab^	13.24 ± 0.05^Aa^

* Different lower case letters in rows and different uppercase letters in columns mean significant differences at probability level of 5%.

Table 3 shows the values of water‐holding capacity in yogurt samples during storage. In all yogurt variations, the WHC values increased significantly (*p *< .05) during storage. The maximum and minimum amounts were recorded on day 21 and on day 1, respectively. Furthermore, this study revealed that the jujube mucilage can reduce the amount of WHC in yogurt. On all days of the storage period, there were significant differences between the control (which exhibited the maximum value) and the samples with mucilage. However, each sample which contained mucilage showed a significant difference (*p *< .05) only on day 1 and day 21 through the timeline of a specific sample considered alone. Several factors including titratable acidity, protein, fat, and storage temperature are known to affect the WHC of yogurt. Moreover, adding special gums which are capable of binding to water can increase the WHC of yogurt samples. Sahan et al. (2008) stated that the addition of the b‐glucan composite does not necessarily change the WHC of nonfat yogurts. However, an insignificant decrease was observed in the WHC values during storage. In another study Doleyres, Schaub, and Lacroix (2005), it was found that the yogurts containing exopolysaccharide‐producing cultures can have a better capacity of storing water which increases over time and results in a lower degree of syneresis. In this context, Ünal, Metin, and Isıklı, (2003) demonstrated that the viscosity and water‐holding capacity can be enhanced due to the increase in the LBG concentration, and it was suggested that the yogurts undergo a decrease in the rate of syneresis.

### 3.6 Viscosity

Table 3 also demonstrates the changes in viscosity among the samples of different treatments over the 21 days of storage. The results show that the viscosity values in all of the samples increased significantly (*p* < .05) throughout storage. The increase was generally proportionate to the concentration of the jujube mucilage. In this manner, the lowest viscosity value was recorded in the control sample at all times, and the highest value was observed in the sample incorporated with 0.2% mucilage. Since the jujube mucilage can bond with free water in the samples, there is an increase in viscosity. According to the findings of Nguyen et al. (2018), the addition of various hydrocolloids to nonfat yogurt has different effects on viscosity, which is dependent on the type of the hydrocolloid being used. Carrageenan and xanthan hydrocolloids at low concentrations (less than 0.02% for xanthan and 0.1% for carrageenan) can significantly increase viscosity, whereas the addition of modified starch can have no effect on viscosity. It is also known that gelatin can reduce the viscosity of yogurt samples. Basiri et al. (2018) reported an increase in viscosity by adding a combination of CMC and FSM to yogurt. However, it was mentioned that the addition of CMC or FSM alone did not have any significant effect on the viscosity of yogurt, compared with the control.

Moreover, according to our results, all treatments caused the highest levels of viscosity in the yogurts after 21 days. The increase in viscosity throughout the storage was also observed in yogurts containing CMC, FSM, or their combinations (Basiri et al., 2018) and in nonfat yogurts containing different concentrations of beta‐glucan (Sahan et al., 2008). The increase in viscosity values during storage might be attributed to the structural rearrangement of proteins and also the alteration of protein–protein connections which make new linkages with the mucilage (Donkor et al., 2007).

### 3.7 Color

From the perspective of consumers, color is a decisive parameter of quality. It can represent the freshness, flavor, and the commercial value of a dairy product. Table 4 demonstrates variations of the color parameters L*, a*, and b* in different samples of yogurt. It was mostly observed that adding the jujube mucilage caused a significant decrease in the L* value (as the lightness parameter of color) compared with the control. Moreover, the addition of mucilage increased the a* value (redness), although the increase was significant only in the first and seventh days of storage. This significant difference was observed between samples containing 0 or 0.1% mucilage and those containing 0.15 or 0.2%.

**Table 4:** Changes in color of yogurt samples during 21 days of storage at 4°C (Mean ± *SD*)

Treatments	Storage time (days)			
	1	7	14	21
*L* *				
JM 0	90.26 ± 0.04^ABca^	90.10 ± 0.15^ABa^	89.53 ± 1.34^Aa^	89.96 ± 0.06^Aa^
JM 0.1	90.70 ± 0.07^Aa^	90.36 ± 0.17^Aa^	89.46 ± 0.10^Aa^	89.84 ± 0.27^Aa^
JM 0.15	89.88 ± 0.25^Ba^	89.30 ± 0.59^BCa^	88.75 ± 0.77^Aa^	88.84 ± 0.21^Aa^
JM 0.2	89.07 ± 0.34^Ca^	89.09 ± 0.27^Ca^	88.38 ± 0.63^Aa^	86.93 ± 1.20^Ba^
*a* *				
JM 0	−1.75 ± 0.32^Ba^	−1.67 ± 0.13^Ba^	−1.42 ± 0.04^Aa^	−1.39 ± 0.16^Aa^
JM 0.1	−1.73 ± 0.03^Bc^	−1.62 ± 0.06^Bb^	−1.34 ± 0.07^Aa^	−1.32 ± 0.11^Aa^
JM 0.15	−1.42 ± 0.01^Ab^	−1.46 ± 0.10^Aab^	−1.32 ± 0.07^Aa^	−1.22 ± 0.53^Aa^
JM 0.2	−1.42 ± 0.17^Ab^	−1.44 ± 0.01^Ab^	−1.22 ± 0.05^Aa^	−1.14 ± 0.15^Aa^
*b* *				
JM 0	2.46 ± 0.23^Aa^	2.71 ± 0.31^Ba^	2.96 ± 0.46^Ba^	2.65 ± 0.09^Ba^
JM 0.1	3.37 ± 0.12^Aa^	3.51 ± 0.10^Aa^	3.74 ± 0.35^ABc^	3.42 ± 0.29^ABa^
JM 0.15	3.25 ± 0.01^Aa^	3.54 ± 0.21^Aba^	3.95 ± 0.08^Aa^	3.99 ± 0.43^Aa^
JM 0.2	3.21 ± 0.79^Aa^	3.63 ± 0.20^Aa^	3.67 ± 0.01^ABa^	3.60 ± 0.27^Aa^

* Different lower case letters in rows and different uppercase letters in columns mean significant differences at probability level of 5%.

Adding jujube mucilage caused a significant increase in the b* parameter of all samples. The control sample showed the lowest b*, whereas higher values were observed in samples incorporated with mucilage. Nonetheless, these differences were not statistically significant between samples containing different percentages of mucilage. In addition, the L* and b* parameters in all treatments did not show significant differences (*p *< .05) through time. However, the a* value increased significantly, except in the control sample. The positive values of a* and the negative values of b* can imply that the corresponding values were in the red and blue ranges of color, respectively. Although the yogurts appeared white to the human eye, the instrument recorded the slight red and blue colors. In general, it can be concluded that the addition of jujube mucilage has been effective on the color factors of yogurt, and, in most cases, it caused the decrease in L* but an increase in a* and b* values. In this context, the changes in the color of yogurt samples could be related to the interaction of polysaccharides with milk proteins such as whey and casein proteins. These interactions can potentially modify the rearrangement of milk proteins, and the accumulation of proteins can trigger the formation of a more integrated structure, along with more intra‐chain bonds. This phenomenon, in itself, can lead to a change in the reflection of light which would consequently cause the samples to appear differently (Aryana, Plauche, Rao, McGrew, & Shah, 2007). Staffolo, Bertola, and Martino (2004) stated that the color parameters did not show significant differences in the experiments when analyzing the properties of yogurts as affected by different dietary fibers through time (*p *< .05). However, lower values of lightness and a discreet brownish color were observed when apple fibers were incorporated into yogurts (Staffolo et al., 2004).

### 3.8 Sensory analysis

Average scores for the flavor of stirred yogurt, either with or without mucilage, differed on day 1 and day 21 during storage (Table 5). It is known that the sensory and nutritional characteristics of yogurt, along with its general features, such as acidity, free fatty acid content, and the amount of flavor compounds, are affected by the initial chemical composition of the milk, the process conditions, the addition of flavorings, and the activity of the starter bacteria involved in fermentation (Bonczar, Wszołek, & Siuta, 2002). As shown in Table 5, the appearance of the samples was not significantly different on days 1 and 7. However, on the 14th day and 21st day, the samples which contained mucilage received a lower score from the sensory evaluators in relation to the appearance, compared with the control sample. No significant difference was observed in the consistency of yogurt samples by sensory evaluators. Moreover, the incorporation of jujube mucilage into yogurt reduced the consumers’ desirability in terms of odor and taste, but these differences were not statistically significant (*p*> .05). The overall acceptance score of samples which contained mucilage was less than the control sample. However, the samples incorporated with jujube mucilage appeared to have acceptable sensory properties. In addition, all of the sensory indices in different yogurt samples decreased during the storage time, although this decrease was not significant compared with day 0 (*p*> .05). In line with our results, Staffolo et al. (2004) reported that the addition of inulin, wheat, and bamboo fibers had no significant effect on the sensory characteristics of yogurt. However, fiber‐rich yogurts received a high score in terms of color, flavor, and texture. Noh, Seo, Lee, and Chang (2013) also demonstrated that the addition of CFE (2%–4%) to yogurt did not significantly affect the aroma, softness, taste, and overall liking scores, but the overall preference score significantly decreased in yogurt samples treated with 6% CFE.

**Table 5:** Sensory properties of yogurt samples during 21 days of storage at 4°C (Mean ± *SD*)*

Treatments	Storage time (days)			
	1	7	14	21
Appearance				
JM 0	3.80 ± 0.44^Aa^	4.00 ± 0.00^Aa^	3.88 ± 0.44^Aa^	3.60 ± 0.54^Aa^
JM 0.1	3.80 ± 0.44^Aa^	3.80 ± 0.44^Aa^	3.80 ± 0.44^Aa^	3.40 ± 0.54^Aa^
JM 0.15	4.00 ± 0.00^Aa^	3.80 ± 0.44^Aa^	3.60 ± 0.54^Aa^	3.40 ± 0.54^Aa^
JM 0.2	3.80 ± 0.44^Aa^	3.80 ± 0.44^Aa^	3.40 ± 0.54^Aa^	3.40 ± 0.54^Aa^
Consistency				
JM 0	3.80 ± 0.44^Aa^	4.00 ± 0.00^Aa^	3.80 ± 0.44^Aa^	3.40 ± 0.54^Aa^
JM 0.1	4.00 ± 0.00^Aa^	3.80 ± 0.44^Aa^	3.80 ± 0.44^Aa^	3.60 ± 0.54^Aa^
JM 0.15	3.80 ± 0.44^Aa^	3.60 ± 0.54^Aa^	3.40 ± 0.54^Aa^	3.40 ± 0.54^Aa^
JM 0.2	3.40 ± 0.54^Aa^	3.40 ± 0.54^Aa^	3.40 ± 0.54^Aa^	3.40 ± 0.54^Aa^
Odor				
JM 0	4.00 ± 0.00^Aa^	3.80 ± 0.44^Aa^	3.80 ± 0.44^Aa^	3.80 ± 0.44^Aa^
JM 0.1	3.80 ± 0.44^Aa^	3.40 ± 0.54^Aa^	3.60 ± 0.54^Aa^	3.40 ± 0.54^Aa^
JM 0.15	4.00 ± 0.00^Aa^	3.40 ± 0.54^Aa^	3.40 ± 0.54^Aa^	3.20 ± 0.44^Aa^
JM 0.2	3.80 ± 0.44^Aa^	3.60 ± 0.54^Aa^	3.60 ± 0.54^Aa^	3.20 ± 0.44^Aa^
Taste				
JM 0	4.20 ± 0.44^Aa^	4.00 ± 0.00^Aa^	3.80 ± 0.44^Aa^	3.80 ± 0.44^Aa^
JM 0.1	4.00 ± 0.44^Aa^	3.80 ± 0.44^Aa^	4.00 ± 0.00^Aa^	3.40 ± 0.54^Aa^
JM 0.15	3.80 ± 0.44^Aa^	3.40 ± 0.54^Aa^	3.60 ± 0.54^Aa^	3.40 ± 0.54^Aa^
JM 0.2	3.60 ± 0.54^Aa^	3.40 ± 0.54^Aa^	3.40 ± 0.54^Aa^	3.20 ± 0.44^Aa^
Overall acceptability				
JM 0	4.00 ± 0.00^Aa^	4.00 ± 0.00^Aa^	3.80 ± 0.44^Aa^	3.80 ± 0.44^Aa^
JM 0.1	3.80 ± 0.44^Aa^	3.80 ± 0.44^Aa^	3.40 ± 0.54^Aa^	3.40 ± 0.54^Aa^
JM 0.15	3.80 ± 0.44^Aa^	3.40 ± 0.54^Aa^	3.40 ± 0.54^Aa^	3.40 ± 0.44^Aa^
JM 0.2	3.60 ± 0.54^Aa^	3.20 ± 0.44^Aa^	3.40 ± 0.54^Aa^	3.20 ± 0.44^Aa^

* Different lower case letters in rows and different uppercase letters in columns mean significant differences at probability level of 5%.

### 3.9 Rheological properties

**Figure 2:**
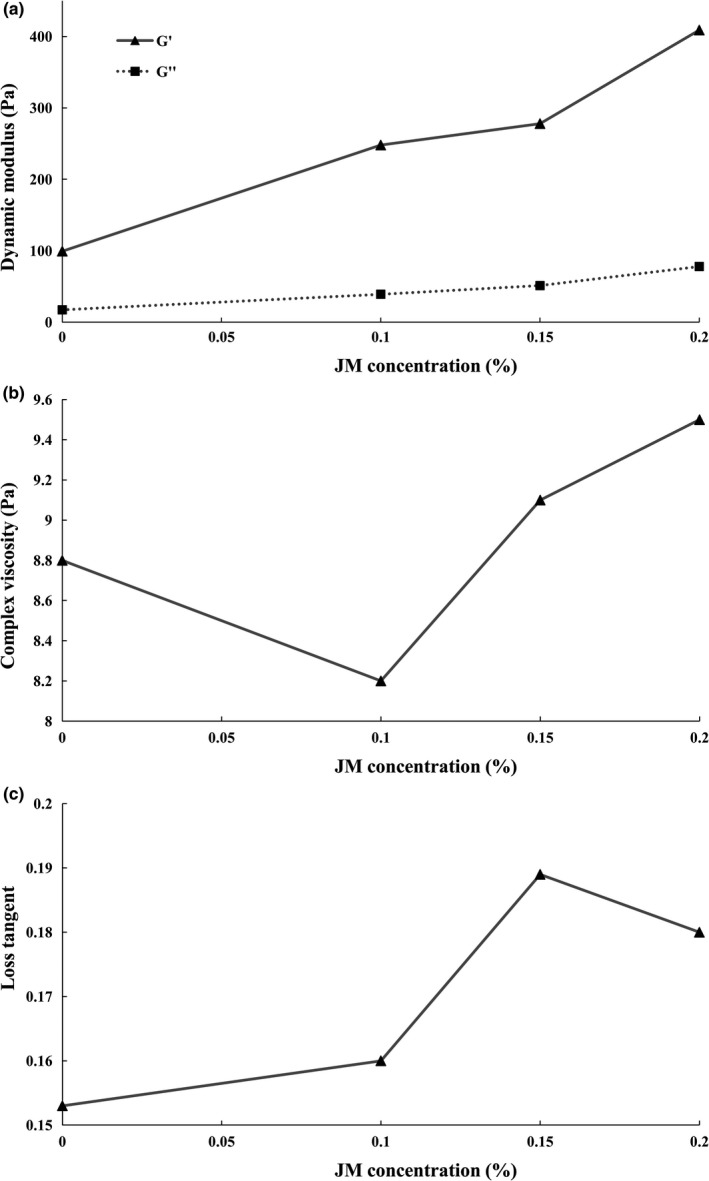
Effects of jujube mucilage concentrations on dynamic modulus (*G*', *G''*) (a), complex viscosity (b), and loss tangent (c) of yogurt samples

Figure 2 shows the rheological attributes of yogurt samples containing various concentrations of mucilage. Stirred yogurt is a viscoelastic material that at higher shear rates, its viscose properties supersede its elasticity. The protein–protein interactions at a molecular level can substantially determine the extent to which the gel‐based structure of yogurts shows elastic properties, whereas weak attractions and interactions between molecules cause its viscose property. The rheological behavior of this gel‐based structure can be described by two factors, namely the storage (*G*') and the loss (*G''*) modules. The storage module represents the elastic behavior, while the loss module describes the viscose manner of the sample. As can be seen in Figure 2(a), the *G*' values of the stirred yogurts in the linear viscoelastic region of all samples, with or without mucilage, are significantly higher than the *G''* values, which indicates that the elasticity of yogurts predominates over their viscose properties. At small strains, based on the “amplitude test,” the *G*' had higher values than the *G''* in all of the stirred yogurts, with or without jujube mucilage, after which the values of *G*'and *G''* crossed over. For stirred yogurts incorporated with 0, 0.1, 0.15, and 0.2% jujube mucilage, the *G*' and *G''* values at the crossover point were 87.99, 189.76, 250.45, and 296.99 Pa, respectively. This implies that, at low strains, all stirred yogurt samples intrinsically showed an elastic nature, whereas increasing the strain caused the destruction of the gel structure until the viscose property superseded the elastic nature. Weak gel‐like structures are mostly indicative of the typical behavior of all yogurts. Moreover, by increasing the percentage of jujube mucilage, the values of the two modules increased. Indeed, by increasing the concentration of mucilage, which is classified as a neutral or nonabsorbent hydrocolloid, a nonelectrostatic interaction can occur between the mucilage and casein micelles, which increases the elasticity of yogurt samples. Relevant to our research, Tudorica, Jones, Kuri, and Brennan (2004) noted that the addition of β‐glucan to milk increased the dynamic modulus in the final state of the yogurt, which was attributed to the interaction between beta‐glucan and casein micelles. Lee and Chang (2016) found that guar gum in yogurt can raise the values of *G*' and *G''*, compared with the control sample. Contrary to our results, Ramirez‐Santiago et al. (2010) reported that yogurts enriched with soluble dietary fiber from *Pachyrhizus erosus* presented lower *G*' and *G''* values in the linear viscoelastic region, compared with the plain sample, but there was a lower flow behavior index (n), a higher consistency index (k), and a higher yield stress than those in the control sample.

Complex viscosity and the loss tangent of stirred yogurt samples, with or without mucilage, are provided in Figure 2(b, c). The complex viscosity, which is a measure of total rigidity, indicated higher values in samples with higher concentrations of mucilage. The addition of mucilage to yogurts resulted in a stabilized gel structure, thereby increasing the integrity of the gel network and hence the complex viscosity. Consequently, the mucilage led to a high degree of firmness in yogurts. As reported by Sendra et al. (2010), the addition of orange fiber led to a stronger structure of gel‐like structures in yogurts which could be attributed to a higher water absorption ability of included fiber, thereby increasing the complex viscosity. Another popular material function to describe the viscoelastic behavior is the loss tangent which indicates the superiority of one of the rheological properties (i.e., either viscose or elastic). This factor is defined as the ratio of the energy that is released (related to the viscose property of the material) to the stored energy (related to the elastic state). A higher value of loss tangent is likely to exhibit more of a liquid‐like behavior than a solid‐like behavior. In the present study, the samples containing mucilage were found to have a higher loss tangent than the control samples. However, the samples incorporated with 0.2% mucilage showed a lower loss tangent value than the samples containing 0.15% jujube mucilage. In fact, it could be stated that at low concentrations of mucilage, the yogurt becomes more inclined to display a quasi‐liquid behavior, whereas higher concentrations of the mucilage can increase the viscose behavior of yogurt, thereby causing a more solid state.

### 3.10 Modeling the rheological behavior

Table 6 indicates the values of a, b, c, and d, as adjusted by the rheological models (Equations 2 and 3). Due to the high value of *R* ^2^ obtained for *G*' (which was at least 0.92), and the one for *G''* (at least 0.98), the models were able to fit well with the experimental data. Steffe (1996) described the *b* parameter as a value between 0.037 and 0.84 in gels and in concentrated solutions, respectively. The current research found a value of *b* in a similar range, as it varied between 0.08 (in samples containing 0.1% mucilage) and 0.89 (in samples containing 0.2% mucilage). Regarding the value of the *a* parameter which ranged from 142.98 (in the control sample) to 498.09 (in yogurts enriched with 0.2% mucilage), all of the stirred yogurt treatments were in the range of concentrated solutions (a: 16.26 pa.s^b^, Steffe (1996)) and the gel (a: 5,626 pa s^b^, Steffe (1996)), although the present data indicate that they were more closer to the values of the concentrated solutions. The values of the *c* parameter varied from 27.89 (in plain yogurt) to 80.20 (in yogurts enriched with 0.2% mucilage), and this can be confirmed in accordance with the report by Steffe (1996), where the *c* value of the samples appeared close to that of the concentrated solutions (27.78). The *d* values ranged from 0.249 (in samples containing 0.2% mucilage) to 0.289 (in plain samples). It was noticeable that the mucilage concentration did have a significant effect on the values of a, b, and c. However, regarding the b and c parameters, the plain yogurt did not show a significant difference compared to the yogurt incorporated with 0.1% mucilage. Staffolo et al. (2004) found similar results in the case of adding dietary fibers to yogurts, which affected their rheological and sensorial attributes.

**Table 6:** Effects of jujube concentrations on dynamic parameters (*a*, *b*, *c*, and *d*) of yogurt samples

Parameter	Treatments			
	JM 0	JM 0.1	JM 0.15	JM 0.2
*a* (pa.s^b^)	142.90	279.04	341.04	498.09
*b* (−)	0.12	0.08	0.79	0.89
*c* (pa.s^d^)	30.99	27.89	55.93	80.20
*d* (−)	0.29	0.28	0.25	0.25
*F* (Hz)	0.05–6.95	0.05–6.65	0.05–6.95	0.10–11.80
Ω = 2pf (rad/s)	0.31–42.54	0.31–70.45	0.31–42.54	0.31–73.77
Adj‐*R* ^2^ (*G*')	0.95	0.92	0.95	0.98
Adj‐*R* ^2^ (*G''*)	0.99	0.99	0.99	0.99

Table 7 shows the results obtained from the adjusted rheological model concerning the experimental data used in Equation 4. Due to the high *R* ^2^ value (0.96–0.99), an appropriate adjustment was made between the model and the data. In addition, as the percentage of mucilage increased, the rate of η* increased from 8.8 to 9.9 pa.s and the dynamic consistency index (a or k*) increased from 79.32 to 225.23. Moreover, the value of n* (or b*) varied from 0.9 to 0.92 among different samples, indicating the viscoelasticity of the above systems, although they were closer to viscose systems. In addition, by adding mucilage, the percentage of the linear viscoelastic region decreased and the yield stress was increased. In this regard, Keogh and O'kennedy (1998) studied the inclusion of milk fat, protein, and hydrocolloids in stirred yogurt. It was found that the starch did not affect the k*, whereas the gelatin and the xanthan/LBG mixture reduced the k* value. However, none of the variables showed a significant effect on the value of the n* parameter.

**Table 7:** Effects of jujube mucilage concentrations on dynamic parameters (k*, n*), linear viscoelastic region, and the yield stress of yogurt samples

Treatments	a(or k*), b(or n*), *R* ^2^	LVE (%)	τ *y* (Pa)	η* (Pa.s)
JM 0	79.32–0.91–0.97	30.30	98.00	8.80
JM 0.1	119.54–0.92–0.98	25.70	104.00	8.20
JM 0.15	139.61–0.90–0.96	18.20	107.00	9.10
JM 0.2	225.23–0.91–0.99	13.80	112.00	9.50

## 4 CONCLUSION

As a natural stabilizer, the jujube mucilage was incorporated into stirred yogurts. Chemical analyses revealed that using the jujube mucilage increased the acidity and proteolysis, but reduced the amounts of acetaldehyde and diacetyl compounds in stirred yogurts, as compared with the control sample. The assessment of physical parameters showed that the stirred yogurt which contained jujube mucilage can be capable of a lower level of syneresis, but a higher viscosity and WHC values. The treated samples showed significantly higher values of dynamic moduli, loss tangent, and complex viscosity, as compared with the control. The jujube mucilage had no significant effect on the sensory characteristics of yogurt, even though it caused yogurts to obtain slightly less scores in terms of overall acceptability. The jujube mucilage has been generally recognized for its beneficial effects on health, besides its function which was revealed here as an improver of physicochemical properties in yogurt. Further research is required to examine the capacities of its commercial applications on other dairy products.

## CONFLICT OF INTEREST

There is no conflict of interest in this paper.

## ETHICAL APPROVAL

This study does not involve any human or animal testing.
